# p130Cas is required for androgen-dependent postnatal development regulation of submandibular glands

**DOI:** 10.1038/s41598-023-32390-1

**Published:** 2023-03-29

**Authors:** Jing Gao, Aonan Li, Shinsuke Fujii, Fei Huang, Chihiro Nakatomi, Ichiro Nakamura, Hiroaki Honda, Tamotsu Kiyoshima, Eijiro Jimi

**Affiliations:** 1 Laboratory of Molecular and Cellular Biochemistry, Division of Oral Biological Sciences, Faculty of Dental Science, Kyushu University, 3-1-1 Maidashi, Higashi-ku, Fukuoka, 812-8582 Japan; 2 Laboratory of Oral Pathology, Division of Maxillofacial Diagnostic and Surgical Sciences, Faculty of Dental Science, Kyushu University, 3-1-1 Maidashi, Higashi-ku, Fukuoka, 812-8582 Japan; 3 Dento-Craniofacial Development and Regeneration Research Center Faculty of Dental Science, Kyushu University, 3-1-1 Maidashi, Higashi-ku, Fukuoka, 812-8582 Japan; 4 Division of Physiology, Kyushu Dental University, 2-6-1 Manazuru, Kokurakita-ku, Kitakyushu, 803-8580 Japan; 5 Department of Rehabilitation, Yugawara Hospital, Japan Community Health Care Organization, 2-21-6 Chuo, Yugawara, Ashigara-shimo, Kanagawa, 259-0396 Japan; 6 Field of Human Disease Models, Major in Advanced Life Sciences and Medicine, Institute of Laboratory Animals, Tokyo Women’s Medical University, 8-1 Kawada-cho, Shinjuku-ku, Tokyo, 162-8666 Japan; 7 Oral Health/Brain Health/Total Health Research Center, Faculty of Dental Science, Kyushu University, 3-1-1 Maidashi, Higashi-ku, Fukuoka, 812-8582 Japan

**Keywords:** Cell polarity, Differentiation

## Abstract

Salivary glands develop through epithelial-mesenchymal interactions and are formed through repeated branching. The Crk-associated substrate protein (p130Cas) serves as an adapter that forms a complex with various proteins via integrin and growth factor signaling, with important regulatory roles in several essential cellular processes. We found that p130Cas is expressed in ductal epithelial cells of the submandibular gland (SMG). We generated epithelial tissue-specific p130Cas-deficient (*p130Cas*^*Δepi–*^) mice and aimed to investigate the physiological role of p130Cas in the postnatal development of salivary glands. Histological analysis showed immature development of granular convoluted tubules (GCT) of the SMG in male *p130Cas*^*Δepi–*^ mice. Immunofluorescence staining showed that nuclear-localized androgen receptors (AR) were specifically decreased in GCT cells in *p130Cas*^*Δepi–*^ mice. Furthermore, epidermal growth factor-positive secretory granules contained in GCT cells were significantly reduced in *p130Cas*^*Δepi–*^ mice with downregulated AR signaling. GCTs lacking p130Cas showed reduced numbers and size of secretory granules, disrupted subcellular localization of the cis-Golgi matrix protein GM130, and sparse endoplasmic reticulum membranes in GCT cells. These results suggest that p130Cas plays a crucial role in androgen-dependent GCT development accompanied with ER-Golgi network formation in SMG by regulating the AR signaling.

## Introduction

Salivary glands are organs formed by epithelial-mesenchymal interactions, and similar to hair, teeth, lungs, and kidneys, their development begins when epithelial cells thicken and invaginate into underlying mesenchymal cells and form through repeated branching^1,2^. The major salivary glands comprise three pairs of glandular organs: the parotid, submandibular, and sublingual glands, which contribute to almost 90% of salivation^3^. A typical gland consists of the glandular epithelium composed of acini, ducts, and myoepithelial cells, surrounded by a stromal matrix. Acini, the secretory end piece, secrete saliva into the lumen of a contiguous ductal network. The duct system of the major salivary glands in humans starts from the acini and progresses to short-intercalated ducts and secretory ducts, which consist of granular cells and striated cells, followed by excretory ducts. The secretory ducts of the parotid gland contain striated ducts alone.

The salivary glands of rodents differentiate postnatally and reach complete maturation several weeks after birth, although the basic structures are constructed during embryonic morphogenesis. The maturation of the submandibular gland (SMG) duct system is evident by the differentiation of granular duct cells from striated duct cells, known as granular convoluted tubules (GCT)^4^. GCT cells differentiate extensively three weeks after birth, and the process partly depends on androgen regulation. After complete maturation of the duct system, the proportion of GCT accounts for 45% to 65% of the gland in males but only 19% to 36% in females^5,6^. GCT cells are characterised by large secretory granules on the subapical side of the cell that contain various biologically active polypeptides, such as epidermal growth factor (EGF), nerve growth factor (NGF), renin, kallikreins (KLK), and proteases^6,7^. Therefore, rodent SMGs provide a useful model for investigating the proliferation and differentiation of epithelial cells in vivo because of their extensive postnatal development.

p130Cas (Crk-associated substrate) is a member of the Cas family and was first identified as a 130 kDa protein that is highly phosphorylated in cells expressing v-Crk (C10 regulator of kinase) and v-Src^8,9^. p130Cas acts as an adapter/scaffold protein by interacting with several binding partners, although it lacks a kinase domain. These interactions are regulated by the phosphorylation of p130Cas which is induced by integrin-mediated adhesion and activation of receptor tyrosine kinase (RTK) or chemokine receptors. p130Cas plays important regulatory roles in several cellular events such as cell mobility, migration, apoptosis, proliferation, and cell cycle^10–14^. Studies using a mouse mammary tumor virus (MMTV)-p130Cas transgenic mouse model overexpressing p130Cas showed that p130Cas plays an important role in mammary gland development^15,16^. Additionally, in the SMG of integrinα3β1 knockout mice, cell polarity is lost and the E-cadherin and fibronectin expression pattern is changed, indicating that integrin signaling is involved in SMG development^17^. As a downstream molecule of the integrin signaling pathway, p130Cas may also be associated with the regulation of salivary gland development.

Previously, we showed that p130Cas is important for ruffle border formation, which is essential for the secretion of acid and proteases for bone resorption by osteoclasts^18^. Osteoclast-specific p130Cas-deficient mice exhibit osteopetrosis in which bone resorption is impaired without ruffled border formation^19^. Furthermore, we recently showed that epithelial cell-specific p130Cas-deficient (*p130Cas*^*Δepi–*^) mice showed hypomineralization in the incisors due to impaired amelogenesis^20^. Salivary gland acinar cells and duct cells are polarised epithelial cells similar to ameloblasts or osteoclasts, both of which secrete various ions and proteins. Because salivary glands develop from ectodermal invagination, similar to teeth and hair follicles, we investigated whether p130Cas is involved in the development of salivary glands using *p130Cas*^*Δepi–*^ mice.

In this study, we showed that p130Cas is expressed in the epithelial duct system, including the GCT, in the SMG. Epithelial cell-specific p130Cas-deficiency caused suppressed androgen receptor (AR) signaling and subsequent development defects in GCTs, which affects the postnatal development of SMG.

## Results

### The expression of p130Cas in the salivary gland

**Figure 1:**
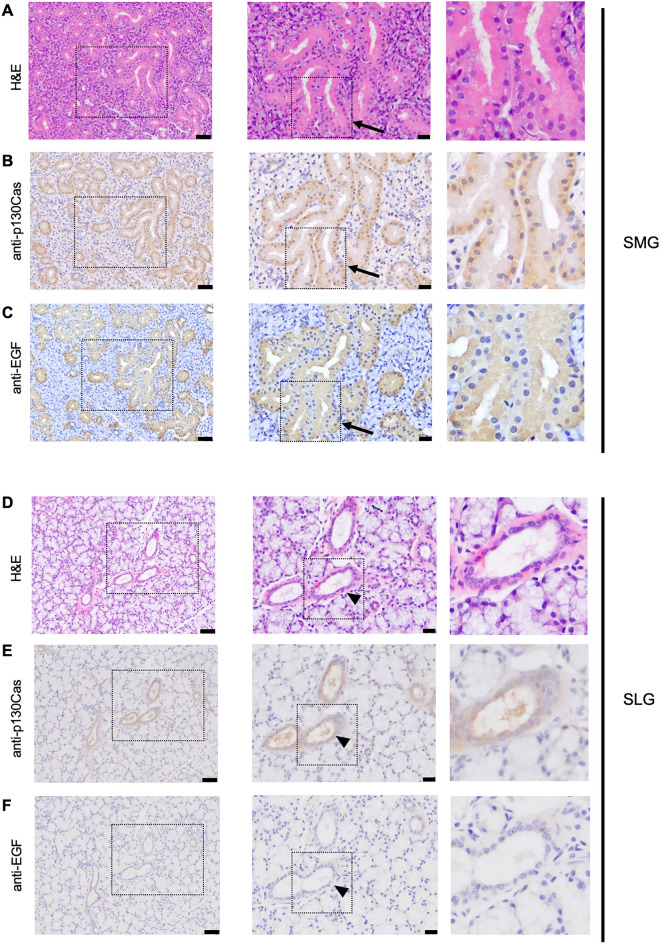
The expression and localization of p130Cas in the salivary gland. Histological analysis of submandibular glands (SMG) (**A**–**C**) and sublingual gland (SLG) (**D**–**F**) from P42 male wild-type mice. Represented images for H&E staining (**A**, **D**), and immunohistochemical staining of SMG and SLG using an anti-p130Cas antibody (**B**, **E**) or anti-EGF antibody (**C**, **F**). Black boxed regions are shown as magnified images. Scale bars = 50 µm (left panel) and 20 µm (middle panel). Arrows indicate the GCT in SMG. Arrowheads indicate ducts in SLG.

To investigate the physiological role of p130Cas in the salivary gland, we first determined whether p130Cas is expressed and its localization in the salivary gland. The mouse SMG and sublingual glands (SLG) are adjacent to each other, and SMGs are the largest salivary glands in mice; therefore, we mainly investigated the SMG and SLG in this study. We performed immunohistochemistry using an anti-p130Cas antibody in postnatal (P) day 42 mice. The expression of p130Cas was observed in ductal cells but not in acinar cells in both the SMG (Fig. 1A–C) and SLG (Fig. 1D–F). Importantly, p130Cas was expressed in the GCT cells which were characterised by the expression of EGF in the P42 male mice (Fig. 1C). The expression of p130Cas was observed only in a few GCT cells in female mice (Fig. S1A). Real-time qPCR analysis showed that the expression level of p130Cas was higher in P42 male mice than female mice which have much less GCTs in SMG (Fig. S1B). These results imply that the expression level of p130Cas correlates with the number of GCTs.

### p130Cas^Δepi–^ mice showed SMG maturation defects due to decreased proliferation and enhanced apoptosis

**Figure 2:**
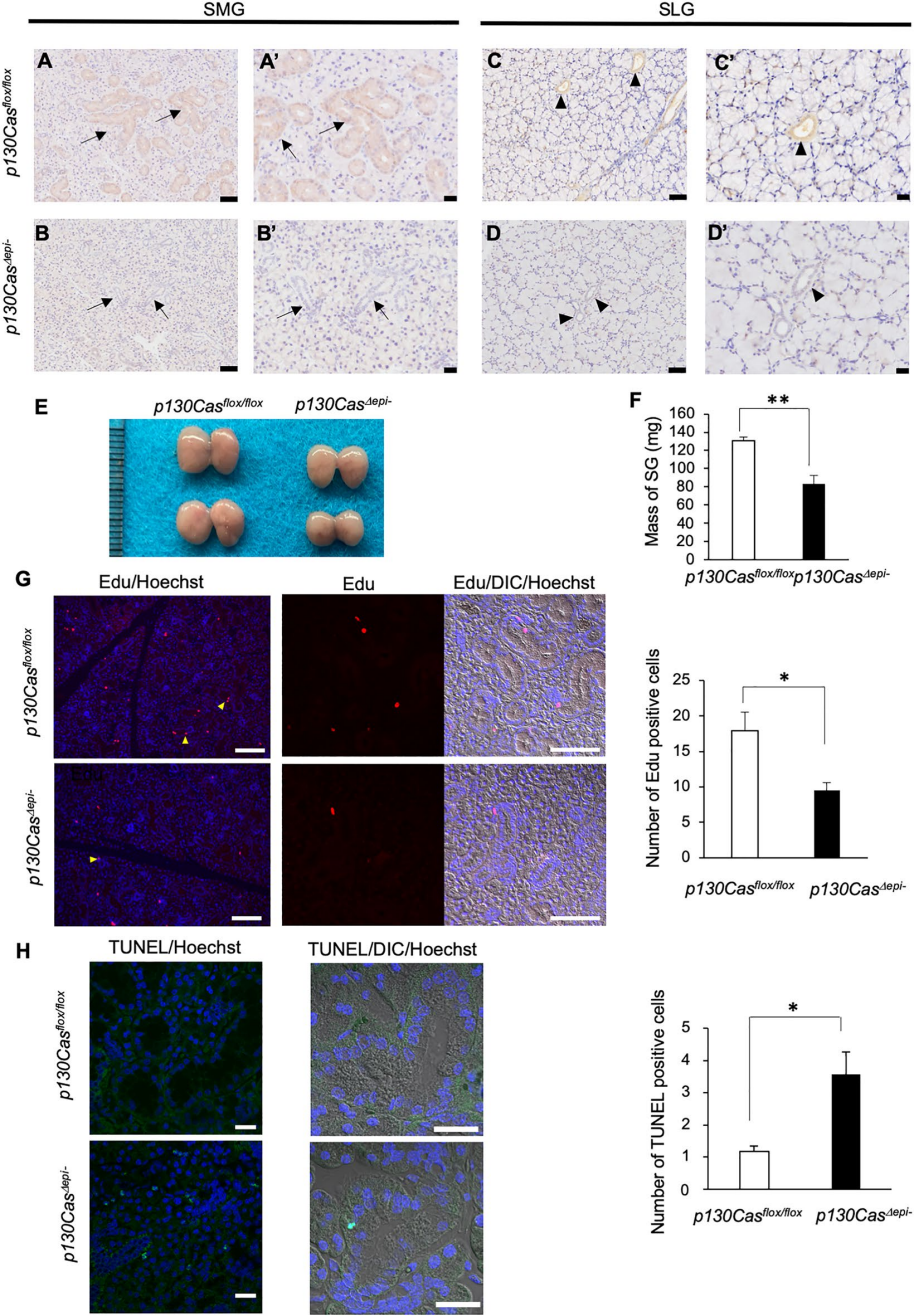
The SMG and SLG phenotypes in *p130Cas*^*Δepi–*^ mice. (**A**–**Dʹ**) Representative images for immunohistochemical staining of SMG (**A**–**B**ʹ) and SLG (**C**–**D**ʹ) from P42 *p130Cas*^*flox/flox*^ and *p130Cas*^*Δepi–*^ mice using an anti-p130Cas antibody. Black boxed regions are shown as magnified images. Scale bars = 50 µm (**A**, **B**, **C**, **D**) and 20 µm (**Aʹ**, **Bʹ**, **Cʹ**, **Dʹ**). Arrows indicate the GCT in SMG. Arrowheads indicate ducts in SLG. (**E**) The gross appearance of the male SMG and SLG in P42 *p130Cas*^*flox/flox*^ and *p130Cas*^*Δepi–*^ mice. (**F**) The total weight of SMG and SLG (salivary gland; SG) from P42 *p130Cas*^*flox/flox*^ and *p130Cas*^*Δepi–*^ mice was measured (*p130Cas*^*flox/flox*^ n = 10 mice, *p130Cas*^*Δepi–*^ n = 8 mice). (**G**) P42 male *p130Cas*^*flox/flox*^ and *p130Cas*^*Δepi–*^ mice were injected intraperitoneally with EdU. Six hours later, mice were sacrificed and the SMG were extracted and processed for tissue sectioning. EdU positive cells were detected using the Click-iT Plus Alexa Fluor Picolyl Azide Toolkit and nuclei were counterstained using Hoechst 33,342. Arrowheads indicate EdU-positive cells. EdU signals, Hoechst 33,342 and DIC (Differential interference contrast) merged images are shown. The number of Edu-positive cells per section was quantified. Scale bars = 100 µm. (**H**) Cellular apoptosis was analyzed via the TUNEL assay and nuclei were counterstained with Hoechst 33,342. TUNEL signals, Hoechst 33,342 and DIC merged images are shown. The number of TUNEL-positive cells per field of view was quantified. Scale bars = 25 µm. Data show the means ± SEM, *P < 0.05, **P < 0.01 versus the corresponding *p130Cas*^*flox/flox*^ value.

Because p130Cas-null mice show embryonic lethality^21^, we generated epithelial cell-specific p130Cas-deficient mice (*p130Cas*^*Δepi–*^)^20^. Keratin 14 (K14) Cre mice were previously used for the study of SMG^22,23^. Furthermore, we confirmed the expression of K14 in GCT cells, basal excretory duct cells, and myoepithelial cells in the SMG (Fig. S2A–D). Immunohistochemistry analysis of P42 SMG and SLG tissue sections showed that the expression of p130Cas was observed in ductal cells including the GCT in *p130Cas*^*flox/flox*^ mice. However, no immune signals were detected in GCT cells in *p130Cas*^*Δepi–*^ mice, even though the expression of p130Cas was observed in excretory duct cells in which K14 was not expressed (Fig. 2A–D; Fig. S2E). These results indicate that p130Cas was successfully deleted in GCT cells.

Next, we extracted and weighed SMG and SLG from P42 male and female mice, as both glands were tightly attached. The gross appearance analysis showed that the weight of SMG in P42 male *p130Cas*^*Δepi–*^ mice were lower than those in *p130Cas*^*flox/flox*^ mice (Fig. 2E, F). However, this phenotype was not observed in the female mice (Fig. S3). Next, we evaluated whether the decrease in SMG mass was caused by reduced cell proliferation or increased cellular apoptosis. For analysis of cell proliferation, we administered P35 male mice with an intraperitoneal injection of Edu (5-ethynyl-2ʹ-deoxyuridine, 50 mg/kg). Six hours later, the SMG was removed, fixed, and progressively dehydrated for tissue sectioning. As shown in Fig. 2G, Edu-labelled cells (red fluorescence) appeared in both the area of acinus and ducts in *p130Cas*^*flox/flox*^ mice. However, the number of Edu-labelled cells was reduced significantly in *p130Cas*^*Δepi–*^ mice, indicating lower cell proliferation in *p130Cas*^*Δepi–*^ mice. On the other hand, TUNEL-positive cells showing green stained nuclei were observed in *p130Cas*^*Δepi–*^ mice, which were almost undetectable in *p130Cas*^*flox/flox*^ mice (Fig. 2H).

**Figure 3:**
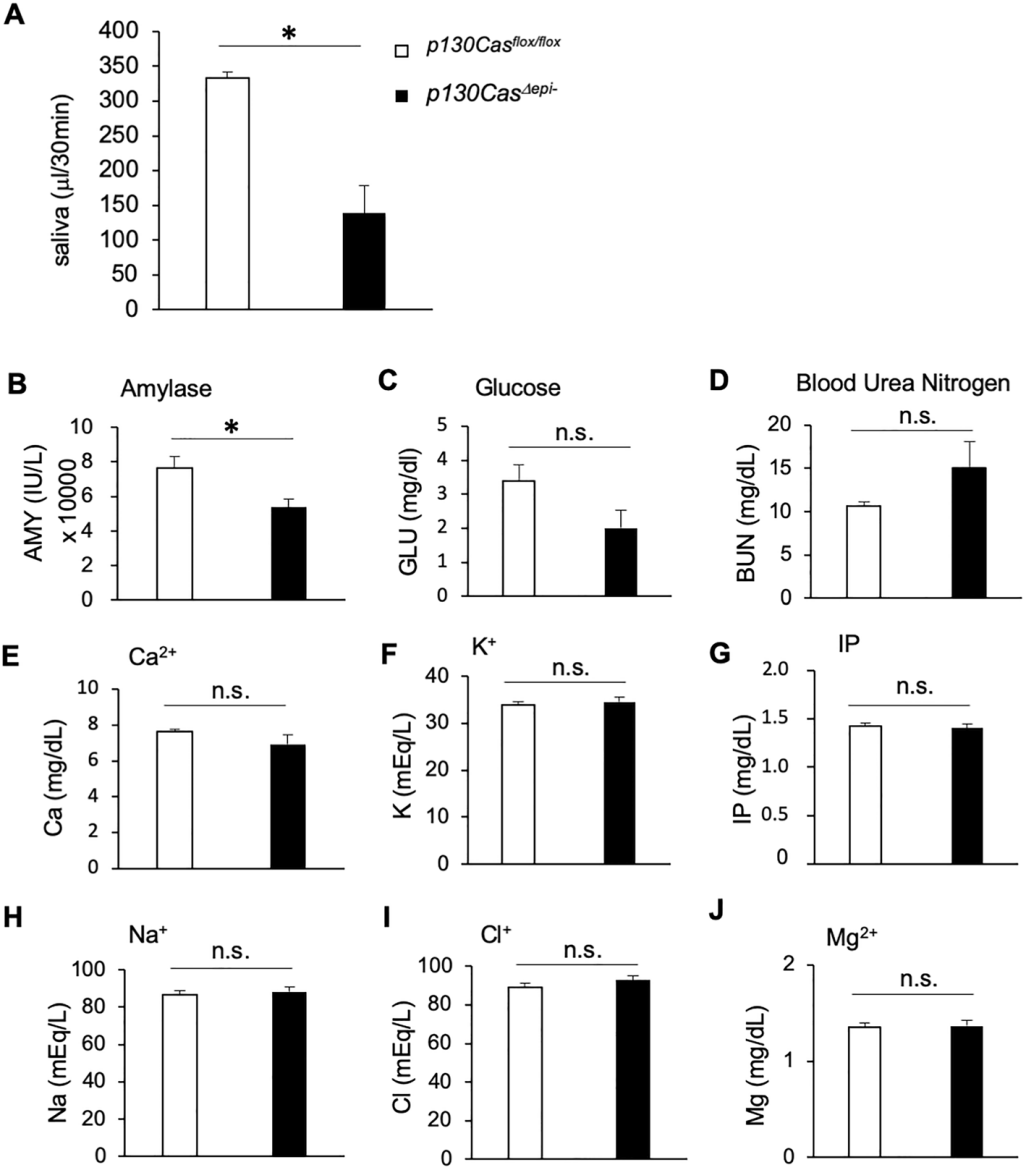
The dysfunction of salivary glands in *p130Cas*^*Δepi–*^ mice. (**A**) The total volume of pilocarpine-induced saliva secretion in 30 min in P35 *p130Cas*^*flox/flox*^ and *p130Cas*^*Δepi–*^ mice. (**B**–**D**) The amounts of organic components of saliva, amylase (**B**), glucose (**C**), and blood urea nitrogen (**D**) were measured. (**E**–**J**) The inorganic component of saliva, Ca^2+^ (**E**), K^+^ (**F**), Na^+^ (**G**), Mg^2+^ (**H**), Cl^–^ (**I**), and IP (inorganic phosphorus) (**J**) were measured. *p130Cas*^*flox/flox*^ n = 10 mice, *p130Cas*^*Δepi-*^ n = 7 mice. Data show the means ± SEM, *P < 0.05 versus the corresponding *p130Cas*^*flox/flox*^ value.

We further analysed the amount and components of saliva secretion. *p130Cas*^*flox/flox*^ and *p130Cas*^*Δepi–*^ mice were intraperitoneally injected with pilocarpine and saliva was collected within 30 min. As shown in Fig. 3A, saliva secretion was reduced by 58% in *p130Cas*^*Δepi–*^ mice compared to that in *p130Cas*^*flox/flox*^ mice. Analysis of saliva components showed that amylase levels were significantly reduced in *p130Cas*^*Δepi–*^ mice, whereas other organic components such as glucose and blood urea nitrogen (BUN) showed no change (Fig. 3B–D). None of the inorganic saliva components, such as Ca^2+^, K^+^, Na^+^, and Cl^–^ were altered in *p130Cas*^*Δepi–*^ mice (Fig. 3E–J). Taken together, the results indicate that the SMG of *p130Cas*^*Δepi–*^ mice showed maturation defects in both morphogenesis and function, likely due to decreased proliferation and enhanced apoptosis.

### p130Cas^Δepi–^ mice displayed maturation defects in the GCT of the SMG

**Figure 4:**
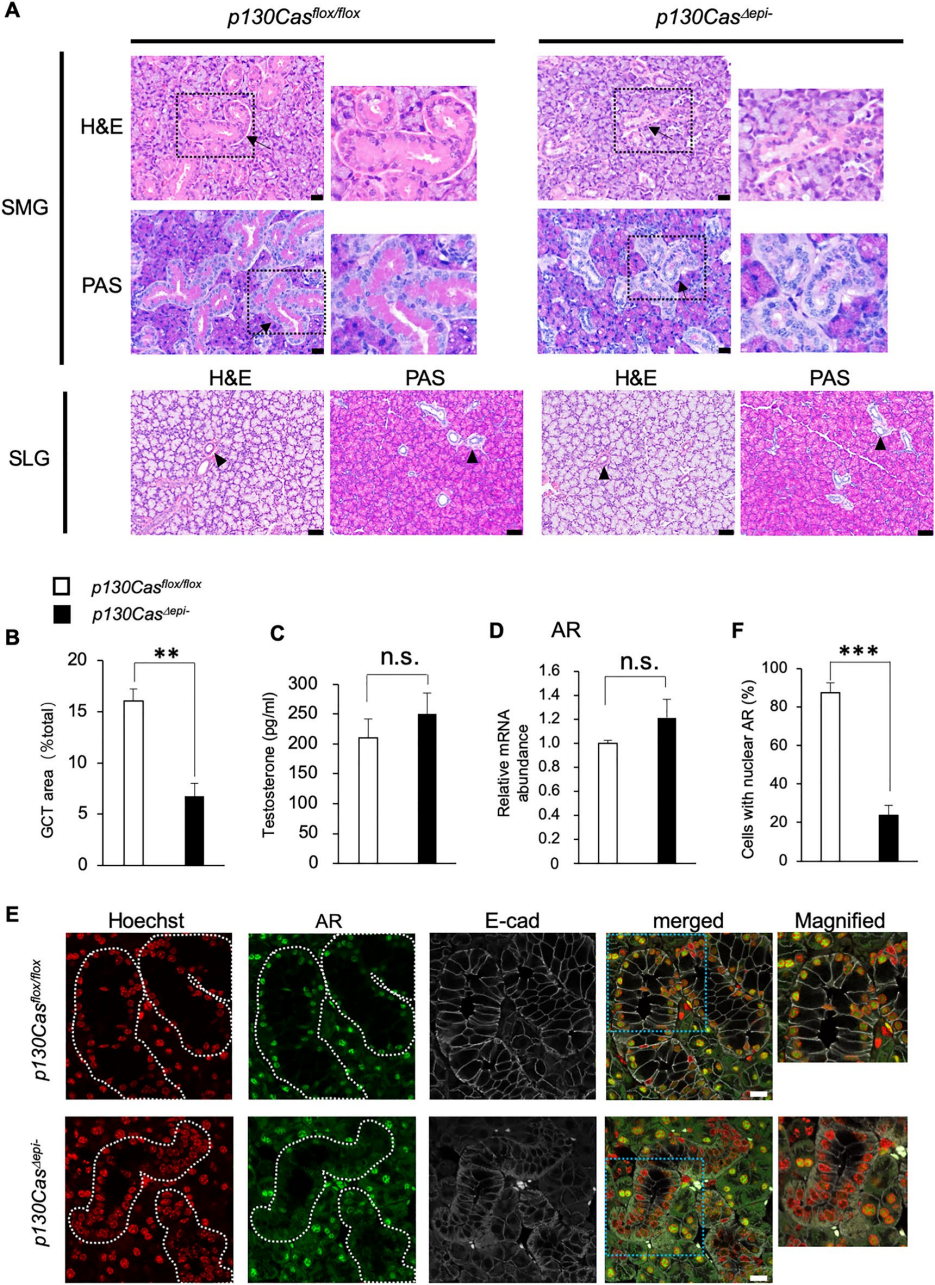
Immature development of the GCT of SMG in *p130Cas*^*Δepi–*^ mice. (**A**) Histological analysis of SMG and SLG in P42 male *p130Cas*^*flox/flox*^ and *p130Cas*^*Δepi*–^ mice. Representative H&E and PAS staining images of paraffin sections. Arrows indicate the GCT in SMG. Arrowheads indicate ducts in SLG. Black boxed regions are shown as magnified images. Scale bars = 20 µm (SMG) and 50 µm (SLG). (**B**) Quantification of the ductal area per total gland area in P42 male *p130Cas*^*flox/flox*^ and *p130Cas*^*Δepi–*^ mice. (**C**) Serum testosterone levels in the P42 male *p130Cas*^*flox/flox*^ and *p130Cas*^*Δepi–*^ mice. (**D**) Quantitative real-time PCR analysis of relative mRNA abundance for the androgen receptor (AR) in SMG in the P42 male *p130Cas*^*flox/flox*^ and *p130Cas*^*Δepi–*^ mice. *p130Cas*^*flox/flox*^ n = 10 mice, *p130Cas*^*Δepi-*^ n = 7 mice. (**E**) Representative immunofluorescence staining of SMG paraffin sections using anti-AR (green) and anti-E-cadherin (gray). Nuclei were counterstained using Hoechst 33,342 (Red). Areas surrounded by white dotted lines indicate GCTs. Blue boxed regions are shown as magnified images. Scale bars = 10 µm. (**F**) The number of AR-positive nucleus of GCTs per field of view was quantified. Data show the means ± SEM, **P < 0.01, ***P < 0.001 versus the corresponding *p130Cas*^*flox/flox*^ value.

We performed histological analyses of the SMG and SLG in P42 male mice. Hematoxylin and eosin (H&E) and periodic acid-Schiff (PAS) staining of SMG sections showed that numerous GCTs were well-developed and convoluted in male *p130Cas*^*flox/flox*^ mice. The cytoplasm of GCT cells was large, filled with eosinophilic contents on the apical side, and many PAS-positive granules on the luminal side (Fig. 4A). The nucleus was located on the basolateral side of the GCT cells. However, in *p130Cas*^*Δepi–*^ mice, the GCTs were less convoluted, and GCT cells were smaller and lacked eosinophilic contents on the apical side and PAS-positive granules on the luminal side. Instead, the nucleus was present at the apical side of the cells (Fig. 4A). Quantitative data showed that the ductal area of the SMG was significantly reduced from 16% in *p130Cas*^*flox/flox*^ mice to 6.7% in *p130Cas*^*Δepi–*^ mice (Fig. 4B). No significant difference was detected in the SLG (Fig. 4A).

Because the development of GCT is androgen-dependent, we analyzed serum testosterone levels and AR mRNA expression in the SMG. No difference was observed between the *p130Cas*^*flox/flox*^ and *p130Cas*^*Δepi–*^ mouse (Fig. 4C,D). Next, we investigated the cellular localization of AR which is important and indicates its signaling activity by immunofluorescence staining using antibody against AR. AR mainly localized in the nucleus of both acinar and GCT cells in *p130Cas*^*flox/flox*^ mice. However, in *p130Cas*^*Δepi–*^ mouse, AR-positive nucleus in GCT were dramatically decreased while the nuclear localization of AR in acinar was not changed (Fig. 4E,F). Based on this result, we have speculated that AR signaling was specifically inhibited in p130Cas-deficient GCT cells. We also found that E-cadherin which mainly localized on plasma membrane in both GCT and acinar cells diffused in cytoplasm of GCT cells in *p130Cas*^*Δepi–*^ mice (Fig. 4E, S4).

**Figure 5:**
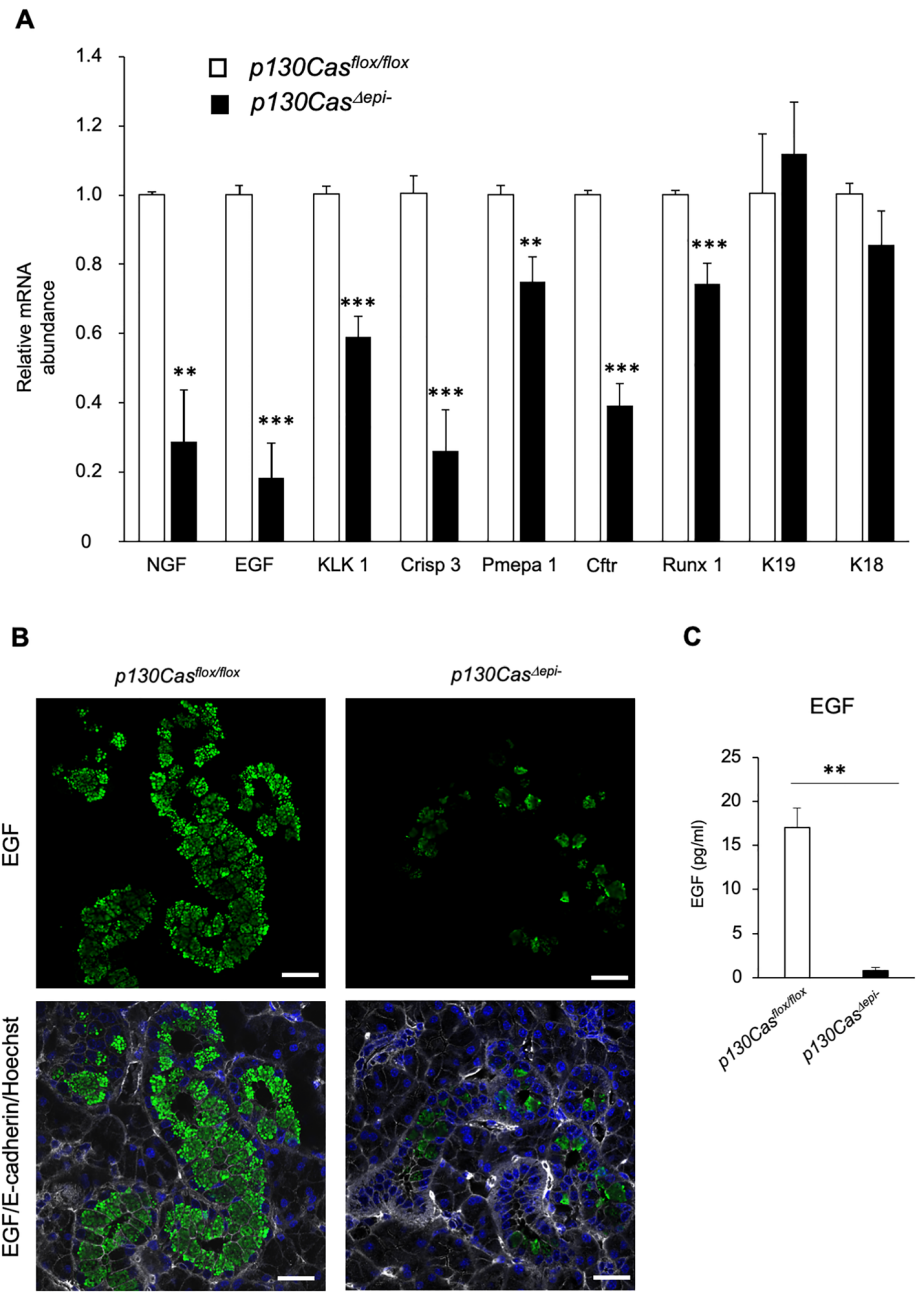
AR signaling is associated with the maturation defects in the GCT of SMG in *p130Cas*^*Δepi-*^ mice. (**A**) Quantitative real-time PCR analysis of relative mRNA abundance in the SMG in P42 male *p130Cas*^*flox/flox*^ and *p130Cas*^*Δepi-*^ mice for NGF, EGF, KLK 1, *Crisp 3*, *Pempa 1*, Ctfr, Runx 1, K19, and K18. (**B**) Representative immunofluorescence staining of SMG paraffin sections using anti-EGF (green) and anti-E-cadherin (grey). Nuclei were counterstained using Hoechst 33,342. Scale bars = 25 µm. (**C**) EGF level in the saliva from male *p130Cas*^*flox/flox*^ and *p130Cas*^*Δepi–*^ mice was measured using an enzyme-linked immunosorbent assay kit. *p130Cas*^*flox/flox*^ n = 9 mice, and *p130Cas*^*Δepi-*^ n = 6 mice. Data show the mean ± SEM, **P < 0.01, ***P < 0.001 versus the corresponding *p130Cas*^*flox/flox*^ value.

In order to examine whether AR signaling was compromised in GCT of *p130Cas*^*Δepi–*^ mouse, we assessed the mRNA levels of several downstream targets of the AR pathway which specifically expressed in GCT cells, such as EGF, NGF, KLK 1^24^, cysteine-rich secretory protein 3 (Crisp 3)^22,25^, prostate transmembrane protein, and androgen-induced 1 (Pmepa 1)^26^, cystic fibrosis transmembrane regulator (Cftr)^27–29^, and Runt-related transcription factor 1(Runx 1)^22,30^. A significant reduction in the mRNA levels of these AR-target genes was observed in the SMG from *p130Cas*^*Δepi–*^ mice (Fig. 5A). EGF, NGF and KLK 1 are also the differentiation maker for GCT, whereas p130Cas deficiency did not alter the expression levels of keratin 19 (K19) which is a marker for excretory ducts, and keratin 18 (K18) which expresses in both acinar and duct cells (Fig. 5A). Furthermore, immunofluorescence staining of SMG sections using antibodies against EGF (green fluorescence) and E-cadherin (grey fluorescence) showed that EGF-positive granules were abundant on the apical side of GCT cells in *p130Cas*^*flox/flox*^ mice (Fig. 5B). In contrast, these secretory granules were dramatically decreased due to p130Cas deletion in GCT cells (Fig. 5B). Further, the amount of EGF in saliva, as measured by enzyme-linked immunosorbent assay (ELISA), was also reduced in *p130Cas*^*Δepi–*^ mice (Fig. 5C). These results indicated that p130Cas deletion in GCT cells caused the suppression of the AR signaling pathway and the subsequent developmental defects of the GCT in SMG.

### Tissue polarity was not compromised by the absence of p130Cas in SMG

**Figure 6:**
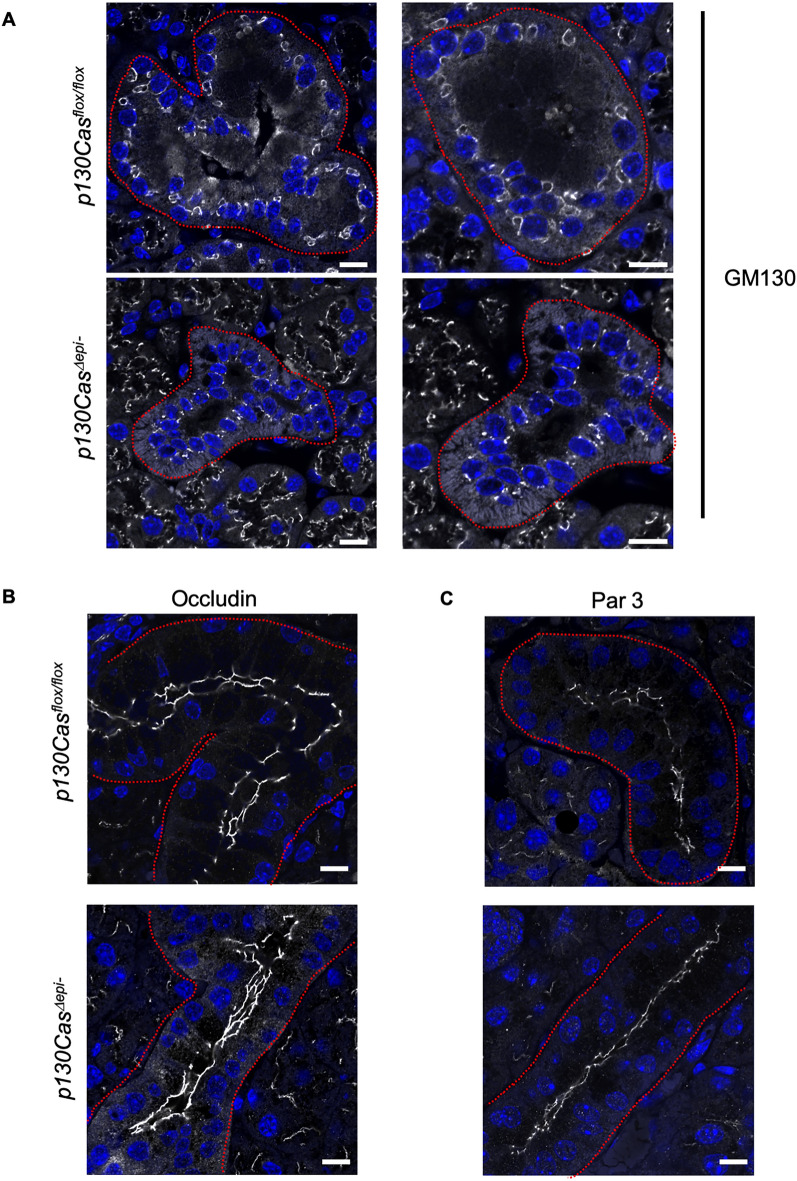
Tissue polarity was not compromised by the absence of p130Cas in the SMG. (**A**–**C**) Representative immunofluorescence staining images of SMG paraffin sections using anti-GM130 (grey) (**A**), anti-occludin (grey) (**B**) and anti-Par3 (grey) (**C**) antibodies. Nuclei were counterstained using Hoechst 33,342. Areas surrounded by dotted lines indicate GCT. Scale bars = 10 µm.

The nucleus which was localized on the basolateral side of control GCT cells was shifted to the apical side in the cells of *p130Cas*^*Δepi–*^ mice (Fig. 4A). We hypothesised that p130Cas may be involved in the modulation of GCT cell polarity which is important for epithelial cells, and investigated the cellular localization of several polarity markers by immunofluorescence staining. The markers included the cis-Golgi matrix protein GM130, which marks Golgi orientation; Par3, which is the master regulator of cell polarity; and occludin, the tight junction proteins which localize near the apical side of GCT cells. p130Cas deficiency resulted in altered intracellular localization of GM130 (Fig. 6A), although no significant difference was observed in the localization of Occludin and Par3 between *p130Cas*^*flox/flox*^ and *p130Cas*^*Δepi–*^ mice (Fig. 6B). These results suggest that p130Cas is not required for the maintenance of GCT cell polarity or tight junction integrity. There may be other reasons for the disturbance in cellular GM130 localization. The nuclear localization in *p130Cas*^*Δepi–*^ mice GCT cells resembles striated duct cells, indicating that these cells failed to differentiate into GCT cells.

### Formation of the ER-Golgi network was impaired by the absence of p130Cas in GCT cells

**Figure 7:**
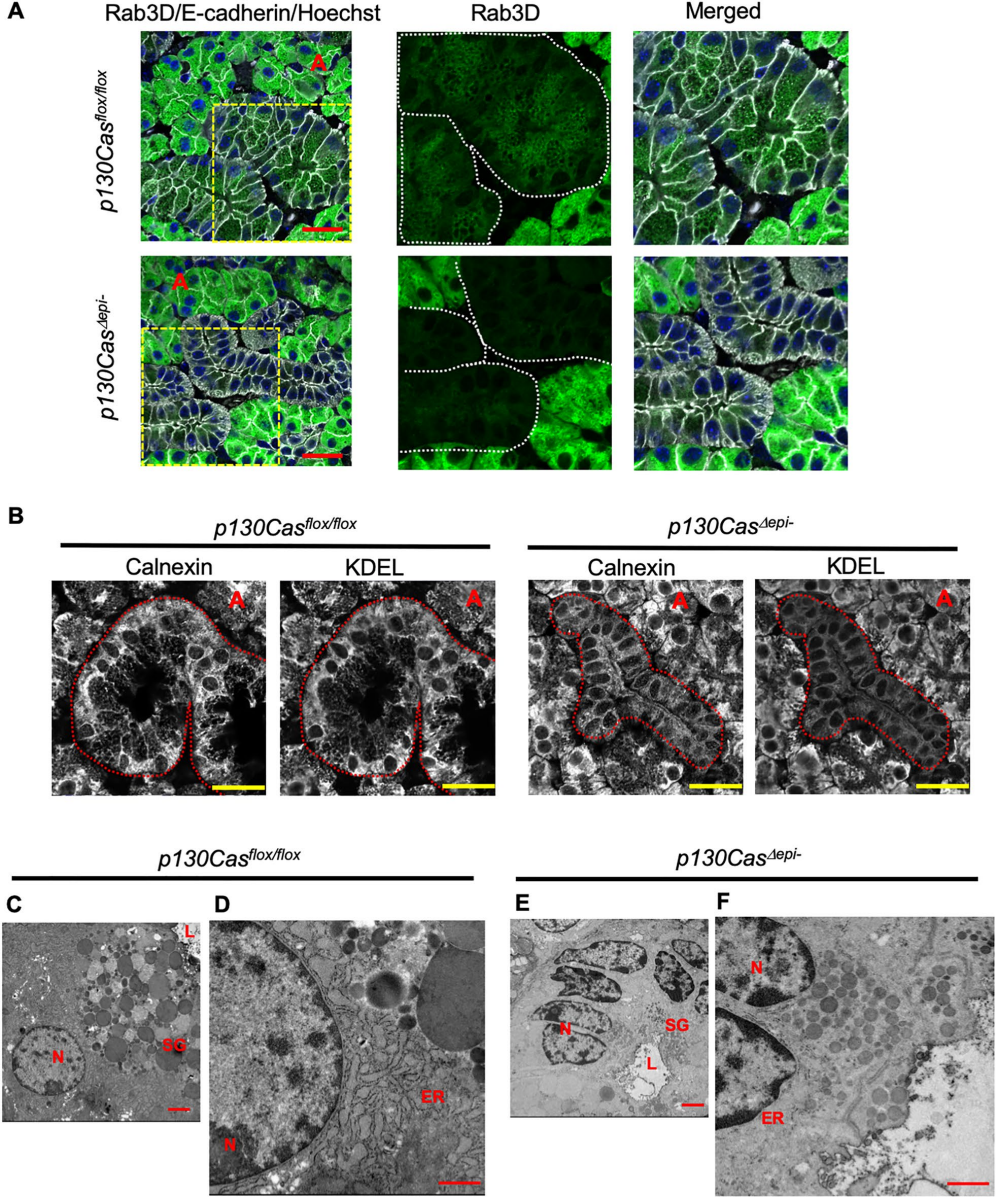
ER-Golgi network was less developed in *p130Cas*^*Δepi-*^ mice. (**A**) Representative immunofluorescence staining images of SMG paraffin sections using anti-Rab3D (green) and anti-E-cadherin (grey). Yellow boxed regions are shown as magnified images. Areas surrounded by white dotted lines indicate GCTs. (**B**) Representative immunofluorescence staining images of SMG paraffin sections using anti-calnexin (grey), and anti-KDEL (grey). Areas surrounded by red dotted lines indicate GCTs. Nuclei were counterstained using Hoechst 33,342. “A” indicates acinar. Scale bars = 25 µm. (**C**–**F**) Representative images of transmission electron microscopy of GCT in male *p130Cas*^*flox/flox*^ and *p130Cas*^*Δepi-*^ mice. Nuleus (N), endoplasmic reticulum (ER), secretory granules (SG), lumen (L). Scale bars = 2 µm (C, E), 1 µm (**D**, **F**).

Immunofluorescence staining using an anti-EGF antibody showed that *p130Cas*^*Δepi–*^ mice GCT cells lacked EGF-positive secretory granules on the subapical side of the cell (Fig. 5B). Next, we verified whether secretory granules were absent in mutant cells or if just EGF was lost in secretory granules. Rab3D, a small GTPase, reportedly localizes to mature secretory granules in the subapical region of epithelial cells^31^. Immunofluorescence staining using anti-Rab3D antibody showed that Rab3D was localized on secretory granules in both acinar and GCT cells and Rab3D was accumulated in the subapical region of the cells. However, Rab3D-positive granules were decreased in GCT cells in *p130Cas*^*Δepi–*^ mice, whereas the cellular localization of Rab3D in acinar cells in *p130Cas*^*Δepi–*^ mice was not altered (Fig. 7A).

Secretory granules bud from the *trans*-Golgi network and release their contents via exocytosis. Endoplasmic reticulum (ER)-to Golgi transport is the first step in the secretory pathway. We have shown that p130Cas deficiency resulted in altered intracellular localization of E-Cadherin and GM130 specifically in GCT cells. Therefore, we examined whether the ER was also affected by the deletion of p130Cas. Immunofluorescence analysis of SMG sections using anti-calnexin (ER marker; grey) and Lys-Asp-Glu-Leu (KDEL; grey) antibodies showed that the ER was well developed in GCT cells and the fluorescence level of them was comparable with that of acinar in *p130Cas*^*flox/flox*^ mice and was accumulated on the basolateral side of the cell and perinuclear regions (Fig. 7B). However, in p130Cas-absent GCT cells, the calnexin and KDEL signals were reduced and became much weaker than that in acinar, although the signals observed in acinar were normal compared with *p130Cas*^*flox/flox*^ mice (Fig. 7B), indicating ER network was compromised in GCTs of *p130Cas*^*Δepi–*^ mice.

To confirm the reduced secretory granules and less-development of ER network, we next visualized male *p130Cas*^*flox/flox*^ and *p130Cas*^*Δepi–*^ mice SMG by transmission electron microscopy (TEM). Figure 7C,D showed a typical GCT cell of *p130Cas*^*flox/flox*^ mouse with a basal euchromatic nucleus and abundant secretory granules occupying the apical three-quarters of the cells. Rich rough ER was located in basal and perinulear regions of the cell^32,33^. In GCT cells of *p130Cas*^*Δepi–*^ mice, rough ER segments were sparse and both reduced number and size of secretory granules were observed, and even some of the cells were absent of granules (Fig. 7E,F). However, no difference in acinar cells between *p130Cas*^*flox/flox*^ and *p130Cas*^*Δepi–*^ mice was observed (Fig. S5A–D). These defects on ultrastructure of *p130Cas*^*Δepi–*^ mice SMG GCT cells supported the results of immunostaining for calnexin and KDEL.

**Figure 8:**
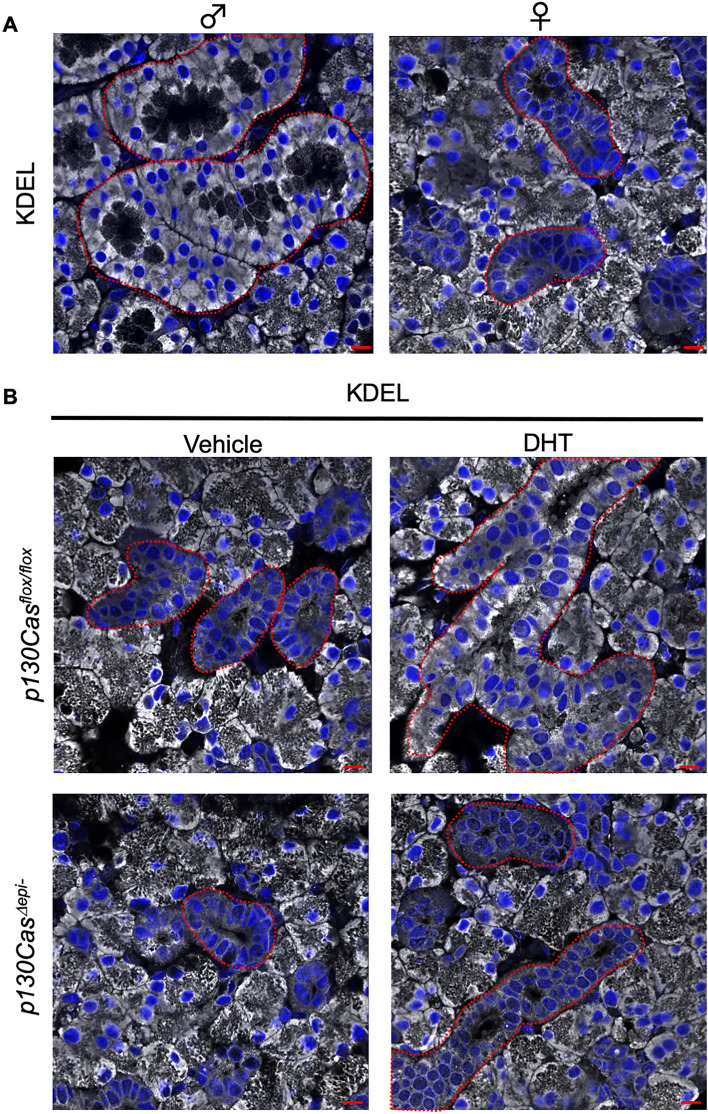
Expansion of ER membrane during GCT differentiation was regulated by AR signaling and compromised in *p130Cas*^*Δepi-*^ mice. (**A**) Representative immunofluorescence staining images of SMG paraffin sections using anti-KDEL (grey) antibody. Nuclei were counterstained using Hoechst 33,342. Areas surrounded by red dotted lines indicate GCTs. Scale bars = 25 µm. (**B**) Representative immunofluorescence staining images of SMG paraffin sections from female *p130Cas*^*flox/flox*^ and *p130Cas*^*Δepi-*^ mice after vehicle or dihydrotestosterone injection using anti-KDEL (grey) antibody. Nuclei were counterstained using Hoechst 33,342. Areas surrounded by red dotted lines indicate GCTs. Scale bars = 25 µm.

We next examined whether the ER network formation was associated with AR signaling in GCT cells. Firstly, we compared KDEL expression in female mouse GCT cells with male mouse by immunofluorescence staining. The KDEL immunofluorescence signal in GCT cells was much stronger in male than in female mice (Fig. 8A). Next, we subcutaneously injected dihydrotestosterone (DHT, 50 mg/kg) to P35 female mice to induce the development of GCT^34^. DHT-induced GCTs in *p130Cas*^*Δepi–*^ mice were not developing as well as that in *p130Cas*^*flox/flox*^ mice as observed in H&E staining of SMG sections (Fig. S5B). Importantly, DHT administration enhanced the KDEL immuno-fluorescence signal in developed GCT cells so that it became even stronger than acinar cells. However, no elevated KDEL immunofluorescence signal was detected in *p130Cas*^*Δepi–*^ mice after DHT administration (Fig. 8B). These results suggest that GCT cells expand the ER membrane and increase ER volume upon AR signaling, but p130Cas-deficient GCT cells failed to expand the ER membrane.

## Discussion

Using *p130Cas*^*Δepi–*^ mice, we showed that p130Cas is required for the postnatal development of GCT in the male SMG. The mutant mice exhibited smaller SMG and impaired salivation function than the control mice. We found that deficiency of p130Cas caused decreased cell proliferation and enhanced apoptosis which may partially explain the smaller SMG. Our results also indicate that p130Cas plays an essential role in both androgen-dependent GCT development and progression of ER-Golgi network formation.p130Cas is involved in the modulation of cell proliferation, which is associated with the activation of Src kinase, extracellular signal-regulated kinase (ERK) 1/2, mitogen-activated protein kinase (MAPK), and Akt pathways^35^. It was reported that SMG epithelial cell proliferation and branching morphogenesis is regulated by multiple growth factors, such as fibroblast growth factors (FGF) and EGF, which stimulates a Erk1/2-dependent downstream signaling^36,37^. Moreover, p130Cas have been shown to play an important role in regulating cell apoptosis including epithelial cell anoikis^38^. Cabodi et al. reported that knockdown of p130Cas using siRNA results in increased apoptosis in mouse TUBO cells^39^, and several other reports showed that overexpression of full-length of p130Cas was antiapoptotic, the proposed mechanisms of which include p130Cas-dependent hyperactivation of FAK, Src, and EGFR pathway signaling proteins (Erk1/2)^40–42^. Pro-apoptotic stimuli trigger dephosphorylation and cleavage of p130Cas, leading to focal adhesion disassembly and translocation of a cleavage product of p130Cas into nuclear to alter the transcription of apoptotic factors^43^. These reports suggest that p130Cas mediates cell proliferation and apoptosis signals as an adapter protein, and that it regulates the transcription of cell proliferation and apoptosis-regulating proteins through changes in localization. Therefore, loss of p130Cas in GCTs may directly or indirectly alter the balance between cell proliferation and apoptosis, leading to suppression of growth and promotion of apoptosis of GCT.

Primary saliva is formed by the salivary acini through two pathways: transcellular and paracellular. The osmotic gradient across the apical plasma membrane attracts water from the cytoplasm via the aquaporin (AQP) 5 water channel^44^. Water may also move from the interstitial space into the lumen via tight junctions. Primary saliva is formed in the acinar lumen along with ion secretions. Our results showed that p130Cas is primarily localized in duct cells, including GCT cells, but not in acinar cells. Therefore, we hypothesised that the cellular localization and expression level of AQP5 in acinar cells from *p130Cas*^*Δepi–*^ mice would not be affected. Immunofluorescence staining using an anti-AQP5 antibody confirmed our hypothesis (Fig. S6). Furthermore, we showed that tight junctions in the acini and ducts showed a normal phenotype in *p130Cas*^*Δepi–*^ mice (Fig. 6). However, p130Cas deficiency reduced the amount of salivary secretion in the mice. This phenotype could be attributed to the less developed GCT which caused the stalling of saliva flow, because the transcellular and paracellular pathways were not affected. This result is supported by the observation that p130Cas deficiency did not significantly change the weight or salivary secretion of SMG in GCT-poor female mice.

Since AR expression is specifically reduced in p130Cas-deficient GCTs, and mRNA levels of AR-regulated target genes specifically expressed in GCT cells, such as EGF, KLK1, Crips3, and Cftr, are reduced, GCT regression in p130Cas^Δepi–^ mice seemed to be due to the inhibition of AR signaling. Furthermore, we also found that no obvious differences were observed in female and male mice before P17, either of which have less-developed GCT (female mice) or no differentiation of GCT (before P17 male mice) (Fig. S7). These results suggest that p130Cas can mediate the AR-induced GCT development, and that the expression of p130Cas correlates with sexual dimorphism in the mouse SMG. The relationship between AR and Src is well studied in prostate cancer, and AR activity is regulated by crosstalk with the Src kinase cascade, implicating activated Src as an important mediator of AR signaling^13,45^. In addition, androgens induce the assembly of a ternary complex comprising AR and Src^46^. Because p130Cas is phosphorylated by Src and interacts with Src, it is possible that it mediates AR signaling by engaging the androgen-AR-Src complex.

Rab3D is a marker of mature secretory granules and the predominant isoform of the Rab3 family in exocrine tissues^30^. Alterations in Rab3D distribution affect secretory functions. In normal acinar cells in the salivary gland, Rab3D localizes primarily to secretory granules in the apical region, whereas Rab3D distribution throughout the cytoplasm was observed in Sjögren’s syndrome, indicating that secretory granules could not be targeted to the apical plasma membrane^47^. GCT cells usually contain many more secretory granules than other duct cells to accommodate their special function of secreting large amounts of various bioactive polypeptides. In *p130Cas*^*Δepi–*^ mice, Rab3D-positive secretory vesicles in GCT cells were decreased compared with control mice, which may cause the observed reduction in EGF and amylase secretion into the saliva.

Specialised cells for secretion, such as pancreatic acinar cells, salivary gland acinar cells, and GCT cells, have a highly developed network of rough ER membranes and a well-organised Golgi apparatus to accommodate the high-synthesis rate, efficient folding, modification, and sorting of proteins^48^. ER membranes have great plasticity. The most impressive example is the differentiation of B lymphocytes into plasma cells^49^. Differentiating lymphocytes dramatically expand their ER membrane, leading to increase in ER volume. Our immunofluorescent staining results with antibodies against two ER markers, calnexin and KDEL and the ultrastructural observation of GCTs, showed that ER membranes were less developed in *p130Cas*^*Δepi*–^ mice than in control mice, indicating that p130Cas deficiency caused a decline in the secretory function of GCT cells. Furthermore, the intracellular localization of GM130 was disturbed in the GCT cells of *p130Cas*^*Δepi–*^ mice, indicating that the Golgi apparatus was also compressed. On the other hand, DHT administration to female mice caused the expansion of rough ER in differentiating GCT cells of control mice which was not observed in *p130Cas*^*Δepi*–^ mice. It was reported that mice with testicular feminisation and AR knockout mice have GCTs with fewer secretory granules^50^. Recently, Hu et al. reported that AR regulates the ER-to-Golgi trafficking pathway, and androgen stimulates ER-Golgi vesicle-mediated transport that promotes protein trafficking in prostate cancer^51^. These provided some evidence for the direct association of AR signaling and ER-Golgi network, although more experiments are needed to clarify the mechanism by which p130Cas-mediated AR signaling regulates formation of ER-Golgi network.

In conclusion, we showed that p130Cas controls cell proliferation and survival in the SMG. p130Cas also acts as a mediator for androgen-dependent differentiation of GCT by regulating AR signaling, including the development of the ER-Golgi network and subsequent secretory vesicle formation. Our results provide new insights into the development of salivary glands and highlight new approaches for the exploitation of therapeutic strategies to promote the recovery of hypofunctioning salivary glands. Furthermore, p130Cas may be associated with the regulation of sexual dimorphism in other diseases, and p130Cas-associated signaling may serve as a new target for drug discovery.

## Methods

### Animals

Epithelial cell-specific p130Cas-deficient (*p130Cas*^*Δepi*–^) mice were generated as described previously^20^. The handling of mice was approved by the Institutional Animal Care and Use Committee of Kyushu University (Approval numbers: A20-138-2, A22-202-0), and all procedures were performed in accordance with the Guidelines for Proper Conduct of Animal Experiments of the Science Council of Japan. All experiments are reported in accordance with ARRIVE guidelines.

### Reagents and antibodies

The antibodies used were as follows: anti-p130Cas (HPA 042282) and Par3 (#07-330) were purchased from Merck Millipore (Billerica, MA). Anti-EGF (ab9695) and anti-AR antibody (ab108341) were purchased from Abcam (Cambridge, UK). Anti-GM130 (#610822) and anti-E-cadherin (#610181) antibodies were purchased from BD Transduction Laboratories (Franklin Lakes, NJ). Anti-keratin 14 (#905304) was purchased from Biolegend (San Diego, CA). Anti-ZO-1 (#21773-1-AP), anti-Rab3D (#12320-1-AP), anti-calnexin (#10427–2-AP), and anti-occludin (#27260–1-AP) antibodies were purchased from Proteintech (Tokyo, Japan). Anti-KDEL (#M181-3) antibody was purchased from MBL (Tokyo, Japan). Pilocarpine hydrochloride was purchased from Fujifilm Wako (Osaka, Japan). Hoechst 33342 was from Merck Millipore.

### Histological analysis and immunohistochemistry

Salivary glands were fixed with 4% paraformaldehyde in 0.1 M phosphate buffer, pH 7.2 and embedded in paraffin. Paraffin sections (5 µm thick) were cut for H&E (Sakura Finetek, Osaka, Japan), PAS, and immunohistochemical staining. H&E and PAS staining were performed as standard procedures.

For immunohistochemistry, after deparaffinization and rehydration, antigen retrieval was performed in an appropriate buffer for each antibody using a decloaking chamber (Biocare Medical, Pacheco, CA). Endogenous peroxide activity was blocked using 1% hydrogen peroxide (Fujifilm Wako) in methanol for 15 min at room temperature, followed by blocking nonspecific protein binding with 1% BSA (Merck Millipore) and 5% goat serum (Merck Millipore) in Tris-buffered saline-Tween 20 (TBS-T). The sections were subsequently incubated with the primary antibody at 4 °C overnight, followed by incubation with a secondary antibody (Histofine Simple Stain MAXPO, Nichirei, Tokyo, Japan) at room temperature for 1 h. Immune complexes were visualised using 3,3ʹ-diaminobenzidine (DAB) substrate solution (Nichirei) and counterstained with haematoxylin.

For immunofluorescence staining, after primary antibody incubation, the immune complexes were visualised using Alexa Fluor 488-conjugated donkey antibodies against rabbit IgG (Thermo Fisher Scientific, Waltham, MA) and AlexaFluor 594-conjugated goat antibodies against mouse IgG (each diluted 1:400) for 60 min at room temperature. The samples were observed using a laser confocal microscope (C2, Nikon, Tokyo, Japan or LSM 510; Carl Zeiss, Jena, Germany).

For transmission electron microscopy (TEM), mice were fixed by perfusion with 4% paraformaldehyde in 0.1 M phosphate buffer, pH 7.2, and then the SMG was extracted, cut into 2 mm^3^ size of block and further fixed with a mixture of 2% paraformaldehyde and 2.5% glutaraldehyde in 0.1 M phosphate buffer, pH 7.2 for overnight, followed by the standard processes for TEM (Tecnai20, Thermo Fisher Scientific).

For DHT administration experiments, P35 female *p130Cas*^*flox/flox*^ and *p130Cas*^*Δepi–*^ mice were subcutaneously injected with vehicle (ethanol) or dihydrotestosterone (DHT, 50 mg/kg) diluted in propylene glycol (Fujifilm Wako). Forty-eight hours later, mice were sacrificed and SMG was extracted, and fixed in 4% paraformaldehyde in 0.1 M phosphate buffer, pH 7.2 for histological analysis.

### RNA extraction and quantitative RT-PCR

Total RNA was extracted from the SMG using a ReliaPrep RNA Miniprep System (Promega, Madison, WI) and reverse-transcribed to cDNA using a High-Capacity cDNA RT Kit (TOYOBO). The resulting cDNA was subjected to quantitative RT-PCR analysis using the KOD SYR qPCR Mix (TOYOBO, Osaka, Japan) in a Takara PCR Thermal Cycler Dice Gradient instrument (Takara Bio, Shiga, Japan). The specific primers used were as follows: EGF F:5ʹ-TGGAACCCAGTGGAATCAC-3ʹ; R:5ʹ-TGGGATAGCCCAATCCGAGA-3ʹ; NGF F:5ʹ-TTTTGATCGGCGTACAGGCA-3ʹ; R:5ʹ-CTGTCACTCGGGCAGCTATT-3ʹ; K18 F:5ʹ-TCAAGATCATCGAAGACCTGAGG-3ʹ; R:5ʹ-GCGCATGGCTAGTTCTGTC-3ʹ; K19 F:5ʹ-GGGGGTTCAGTACGCATTGG-3ʹ; R:5ʹ-GAGGACGAGGTCACGAAGC-3ʹ; *Pmepa 1* F:5ʹ-TGGAGTTCGTGCAAATCGTG-3ʹ; R:5ʹ-TCCGAGGACAGTCCATCGTC-3ʹ; *Crisp 3* F:5ʹ-ACAGTGGCCATTATCCAAGCA-3ʹ; R:5ʹ-GCATGTAGCTAGGCAACGTTTT-3ʹ; KLK 1 F: 5ʹ-CACCCGTCAAATATGAATACCCA-3ʹ; R: 5ʹ-TAGGGCCCCATGATGTGATAC-3ʹ; Cftr F: 5ʹ-CTGGACCACACCAATTTTGAGG-3ʹ; R: 5ʹ-GCGTGGATAAGCTGGGGAT-3ʹ; Runx 1 F: 5ʹ-CTGCAACAAGACCCTGCCCATCGCTTTC-3’; R: 5’-CTCCGCCCGACAAACCTGAGGTCGT-3’; p130Cas F: 5’-CCAAAGCCCTCTATGACAATGT-3’; 5’-CTTGAGGCGGTTACCAGGC-3’; *GAPDH* F:5ʹ-AGGTCGGTGTGAACGGATTTG-3ʹ; R:5ʹ-TGTAGACCATGTAGTTGAGGTCA-3ʹ; Androgen Receptor F:5ʹ-CTGGGAAGGGTCTACCCAC-3ʹ; R:5ʹ-GGTGCTATGTTAGCGGCCTC-3ʹ.

### Analysis of cell proliferation and apoptosis

Five-week-old mice were intraperitoneally injected with 50 mg/kg EdU (Tokyo Chemical Industry, Tokyo, Japan). Six hours later, the salivary glands were extracted, fixed with 4% paraformaldehyde, and embedded in paraffin. Paraffin sections (5 µm thick) were processed to detect Edu-positive cells, and cell proliferation was analysed using the Click-iT Plus Alexa Fluor Picolyl Azide Toolkit (Thermo Fisher Scientific). For analysis of cellular apoptosis, TUNEL staining was performed using the In situ Apoptosis Detection Kit (Takara Bio) according to the manufacturer’s instructions.

### Measurement of serum testosterone levels

Serum testosterone levels were measured using an ELISA kit according to the manufacturer’s instructions (Arbor Assays LLC).

### Measurement of saliva secretion and saliva component analysis

Six-week-old mice were anaesthetised intraperitoneally with medetomidine hydrochloride (0.75 mg/kg body weight, Kyoritsu Seiyaku, Tokyo, Japan), midazolam (4 mg/kg, Fuji Pharma, Tokyo, Japan), and butorphanol tartrate (5 mg/kg, Meiji Seika Pharma, Tokyo, Japan). Saliva secretion was stimulated via an intraperitoneal injection of pilocarpine (5 mg/kg body weight). Small blocks of absorbent cotton were placed in the mouth of the mice to absorb saliva, and the cotton was changed after 2 min. The amount of saliva was determined gravimetrically.

For saliva component analysis, saliva was collected using a microcapillary tube (Hirschmann, Germany), and the components were analysed by ORIENTAL YEAST CO., LTD (Tokyo, Japan). Briefly, amylase activity was measured using the JSCC transferability method. The amount of glucose was measured using the hexokinase-glucose 6 phosphate dehydrogenase (HK-G6PDH) method. The concentrations of Na^+^, Ca^2+^, K^+^, Cl^–^, Mg^2+^, and IP (inorganic phosphorus) were measured using an ion-selective electrode and enzymatic methods, respectively. The amount of BUN was analysed using the urease-glutamate dehydrogenase (GLDH) test.

### Statistical analysis

Quantitative data are shown as the mean ± SEM. Comparisons were performed between two groups using the unpaired Student’s *t *test, and analyzed using GraphPad Prism 9.

## Supplementary Information

Supplementary Information.

## Data Availability

All relevant data is contained within the manuscript.
