# Supplementary material for: p130Cas is required for androgen-dependent postnatal development regulation of submandibular glands
Source: Sci Rep. 2023 Mar 29;13:5144. doi: 10.1038/s41598-023-32390-1 (PMC10060253; doi:10.1038/s41598-023-32390-1)
Supplement: Supplementary file 1 — Supplementary Information. [file 41598_2023_32390_MOESM1_ESM.pdf]

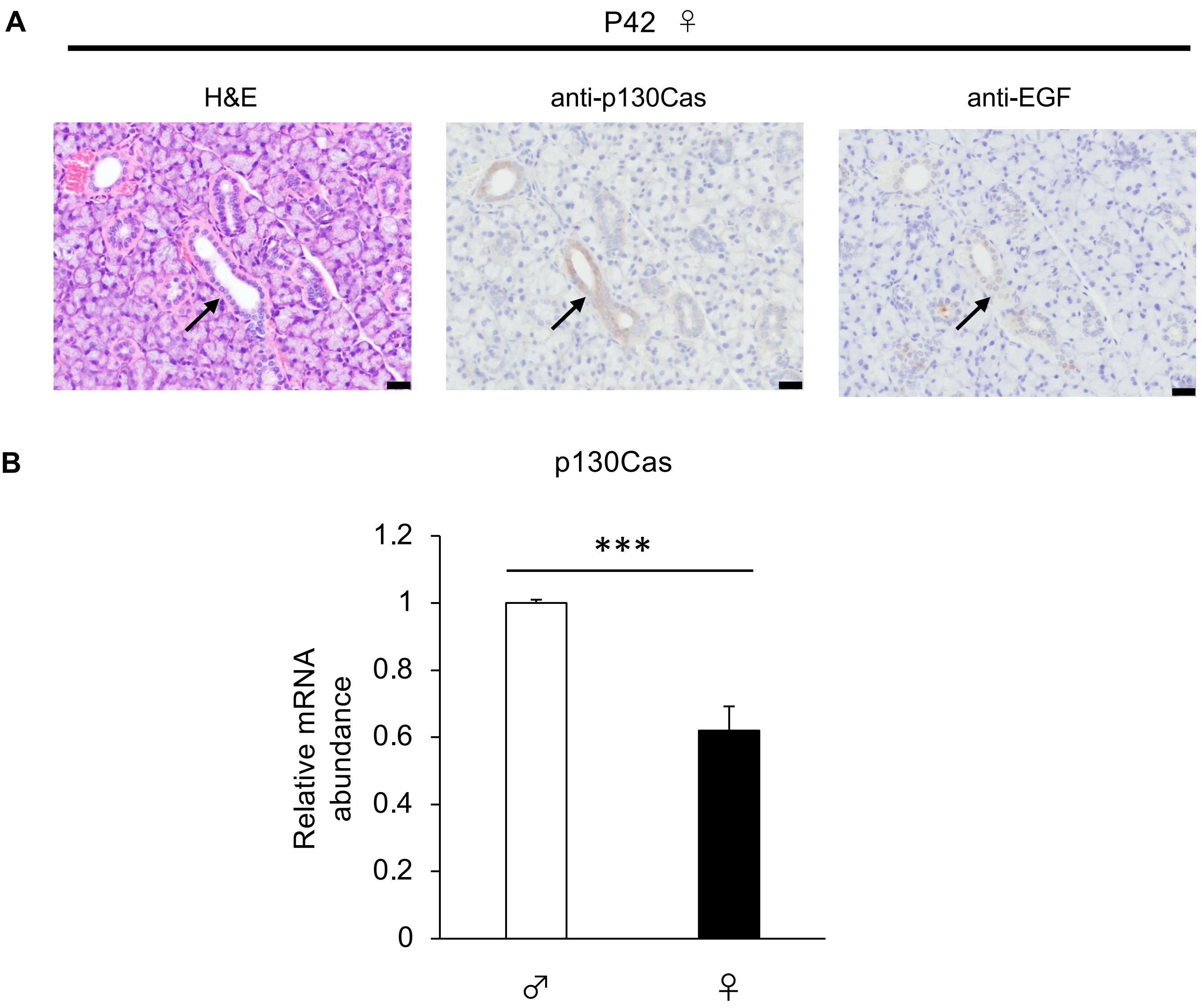

Fig. S1. **(A)** Histological analysis of submandibular glands (SMG) from P42 female wild-type mice. Represented images of hematoxylin and eosin (H&E) staining, immunohistochemical staining of SMG using anti-p130Cas antibody and anti-EGF antibody. Scale bars, 20 μm. **(B)** Quantitative real-time PCR analysis of relative mRNA abundance for p130Cas in the SMG in P42 male and female mice. Male n = 3 mice, female n = 3 mice. Data show the means ± SEM, ****P* < 0.001 versus the male value.

Supplementary Fig. 1. Gao & Li et al.

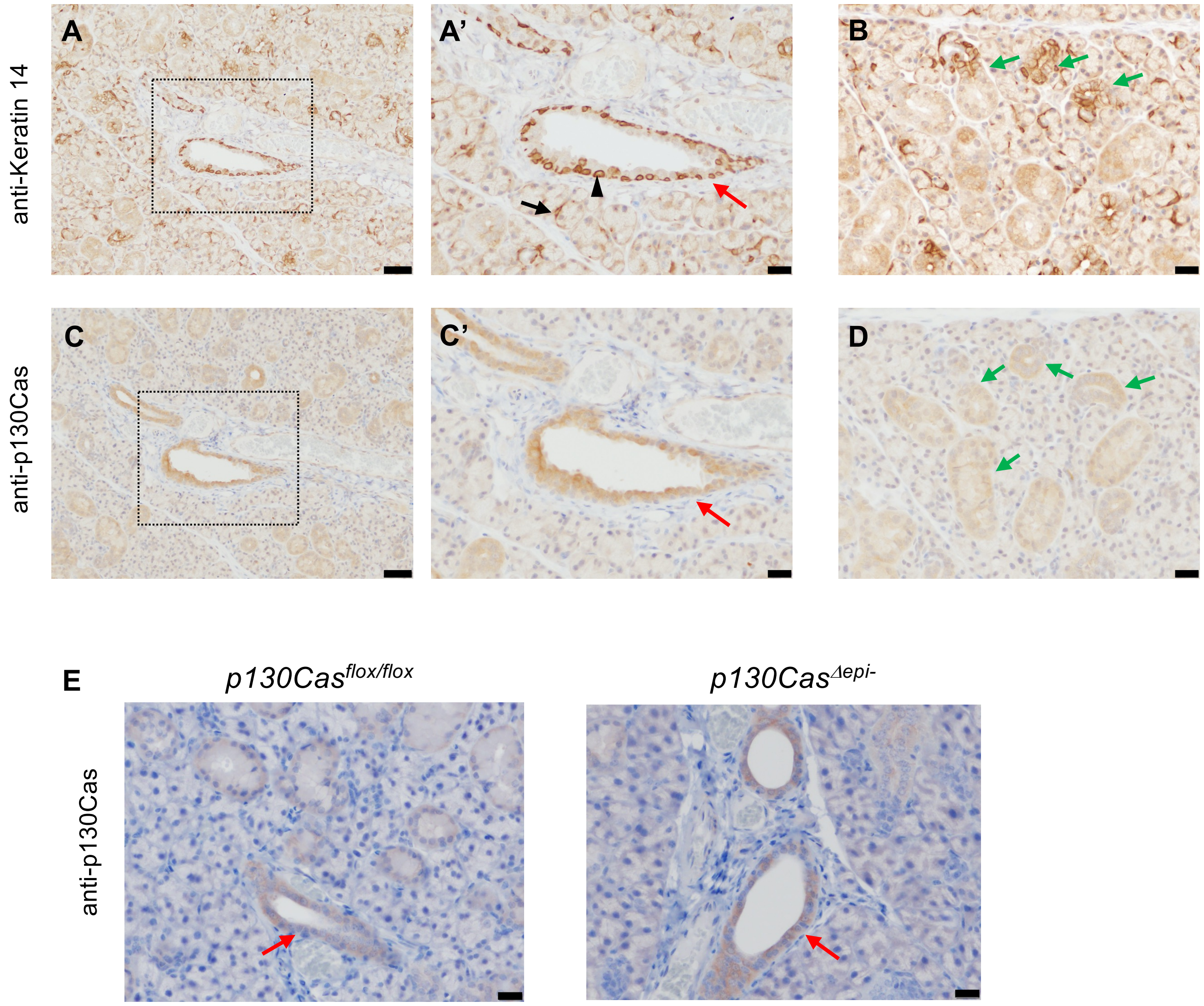

Fig. S2. **(A-D)** Represented images of immunohistochemical staining of SMG from P28 male wild-type mice using anti-keratin 14 antibody (A, A', B) and anti-p130Cas antibody (C, C', D). Black arrowhead indicates basal excretory duct cell. Arrow indicates myoepithelial cell. Green arrows indicate GCT. Scale bars, 20 µm (A, C) and 50 µm (A', B, C', D). **(E)** Represented images of immunohistochemical staining of SMG from P42 *p130Cas*$^{fl/fl}$ and *p130Cas*$^{\Delta epi-}$ mice using anti-p130Cas antibody. Red arrows indicates excretory duct. Scale bars, 20 µm.

Supplementary Fig. 2. Gao & Li et al.

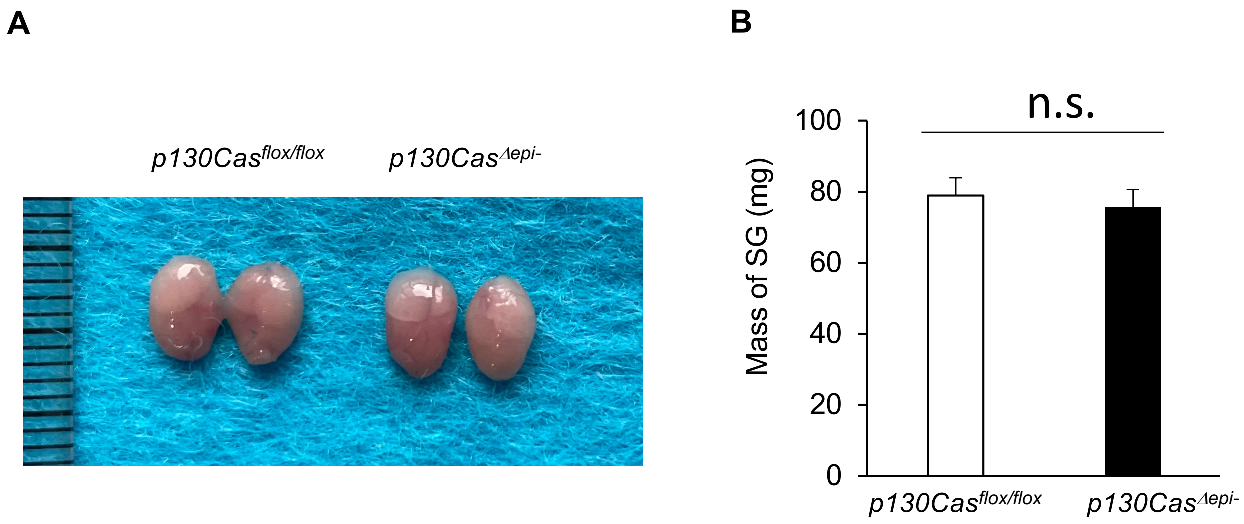

Fig. S3. **(A)** The gross appearance of the female SMG and SLG in P42 *p130Cas*$^{fl/fl}$ and *p130Cas*$^{\Delta epi-}$ mice. **(B)** The total weight of SMG and SLG (salivary gland; SG) from p42 *p130Cas*$^{fl/fl}$ and *p130Cas*$^{\Delta epi-}$ mice was measured (*p130Cas*$^{fl/fl}$ n = 9 mice, *p130Cas*$^{\Delta epi-}$ n = 6 mice).

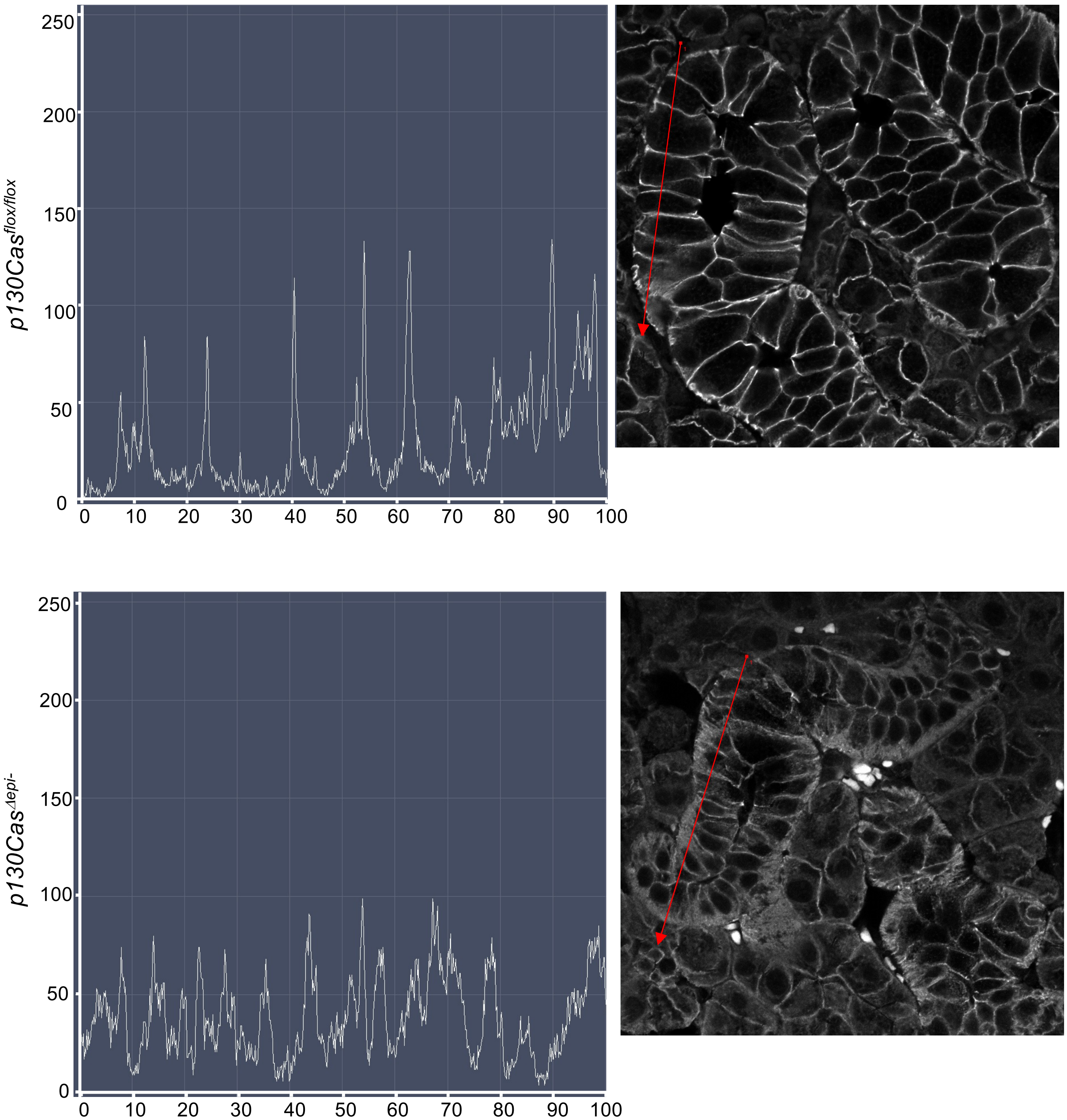

Fig. S4. The intensity of E-cadherin on the red arrow across the GCT was measured using ZEN lite software (Carl Zeiss) to show the plasma membrane or cytosol localization of E-cadherin.

Fig. S4. Gao & Li et al.

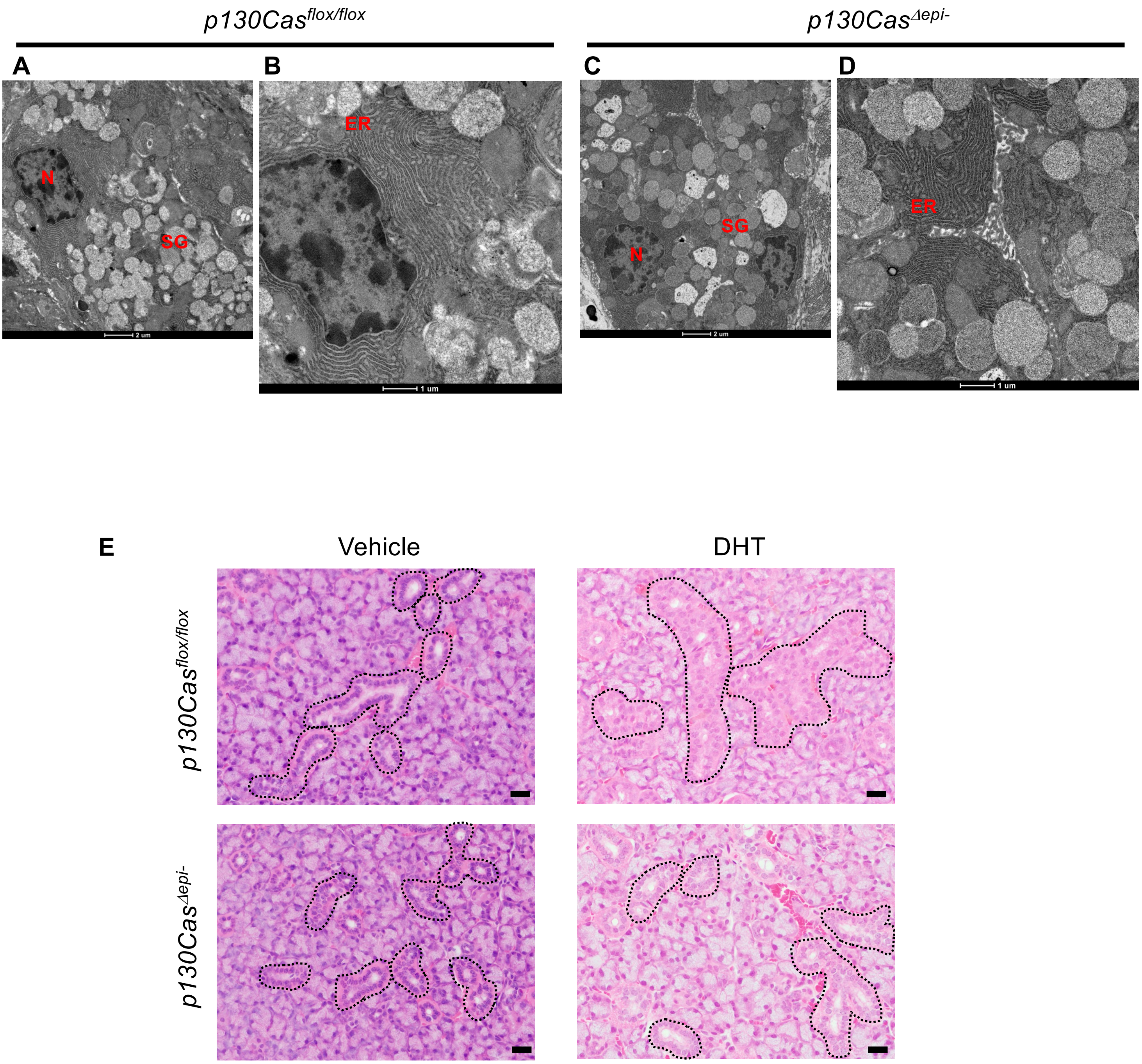

Fig. S5. **(A-D)** Representative images of transmission electron microscopy of acinar cell in male *p130Cas*$^{flox/flox}$ and *p130Cas*$^{\Delta epi-}$ mice. Nuleus (N), endoplamic reticulum (ER), secretory granules (SG). Scale bars = 2 µm (A , C), 1 µm (B, D). **(E)** Representative H&E staining images of SMG paraffin sections from female *p130Cas*$^{flox/flox}$ and *p130Cas*$^{\Delta epi-}$ mice after vehicle or dihydrotestosterone injection. Areas surrounded by black dotted lines indicate GCT. Scale bars = 20 µm.

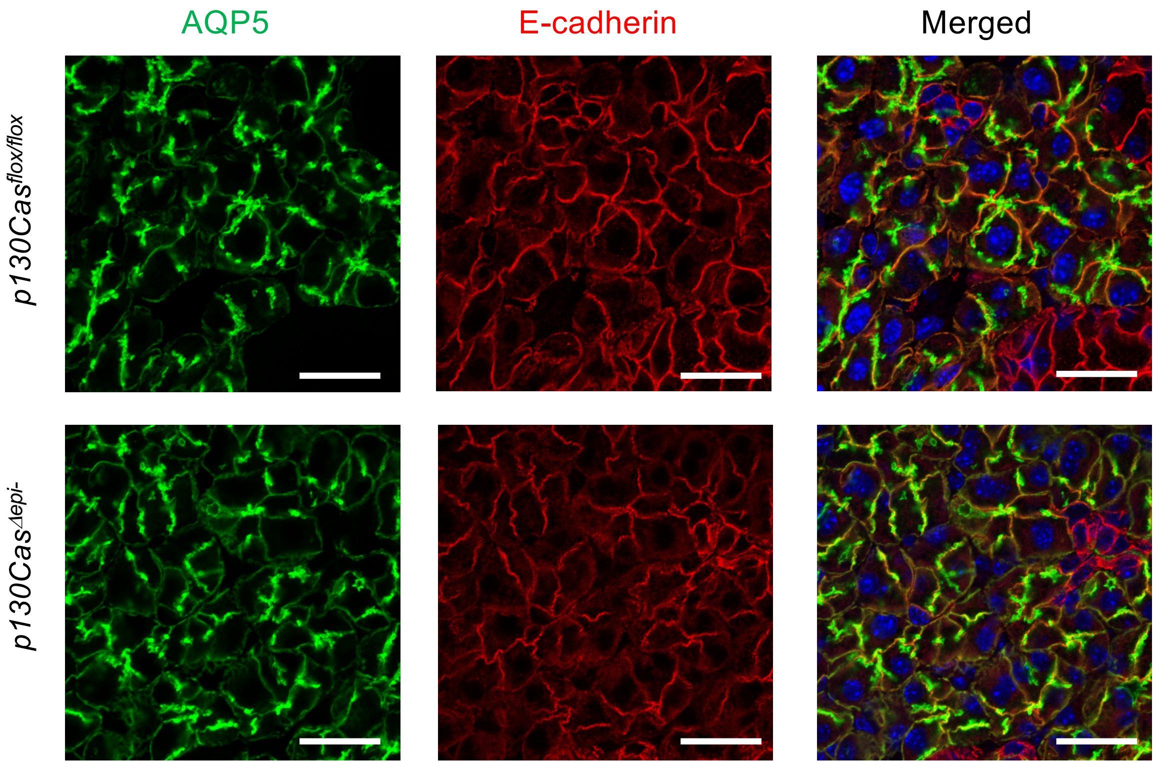

Fig. S6. Represented immunofluorescence staining of SMG paraffin sections using anti-AQP5 (green) antibody and anti-E-cadherin (red) antibody. Nuclei were counterstained with Hoechst 33342. Scale bars, 25 μm.

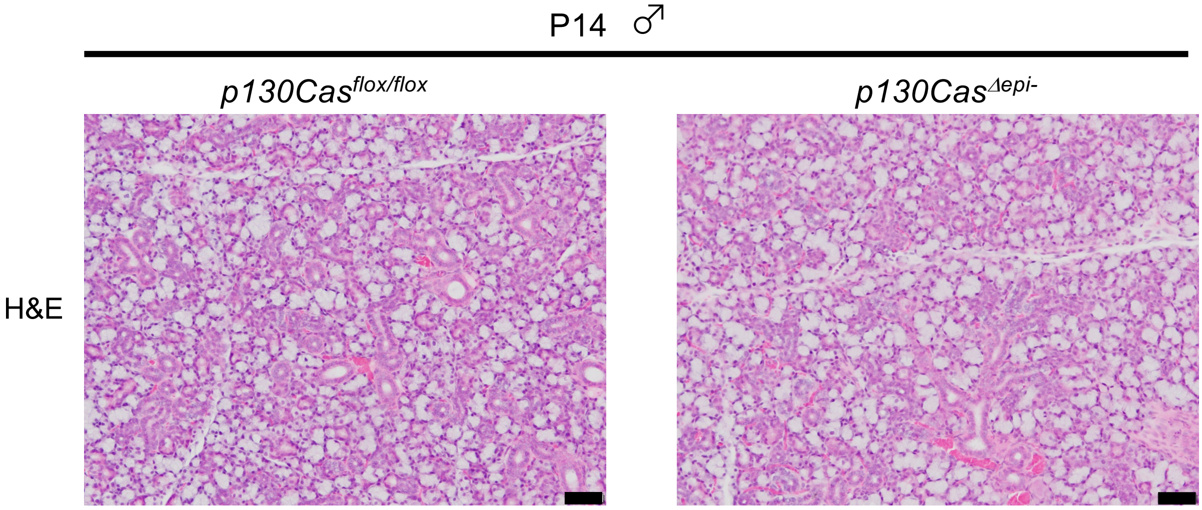

Fig. S7. Histological analysis of submandibular glands (SMG) from P14 male *p130Cas*$^{fl/fl}$ and *p130Cas*$^{\Delta epi-}$ mice. Represented images of H&E staining. Scale bars, 50 μm.
